# Adolescent physical activity, sedentary behavior and sleep in relation to body composition at age 18 years in urban South Africa, Birth-to-Twenty+ Cohort

**DOI:** 10.1186/s12887-020-02451-9

**Published:** 2021-01-11

**Authors:** Lisa K. Micklesfield, Sara K. Hanson, Felipe Lobelo, Solveig A. Cunningham, Terryl J. Hartman, Shane A. Norris, Aryeh D. Stein

**Affiliations:** 1 MRC/Wits Developmental Pathways for Health Research Unit, Faculty of Health Sciences, University of the Witwatersrand, Johannesburg, South Africa; 2 Doctoral Program in Nutrition and Health Sciences, Laney Graduate School, Emory University, Atlanta, GA USA; 3 Hubert Department of Global Health, Rollins School of Public Health, Emory University, Atlanta, GA USA; 4 Department of Epidemiology, Rollins School of Public Health, Emory University, Atlanta, GA USA

## Abstract

**Background:**

Adolescence is marked by a decline in physical activity, rapid physical growth and changes in body composition, which have been linked to body composition. Prospective data on these associations are rare, particularly in Africa.

**Aim:**

The aim of this study was to examine the association of longitudinal patterns across adolescent in physical activity, sedentary behavior and sleep, with anthropometry and body composition at age 18 years in urban South Africa.

**Methods:**

We analyzed data from the Birth-to-Twenty Plus Cohort (Bt20+), a longitudinal study of children born in 1990 in Soweto-Johannesburg, South Africa. We used general linear models to investigate the association of adolescent (ages 12 to 18 years) longitudinal trends in physical activity, sedentary behavior and schoolnight sleep and overall physical activity patterns, with body mass index (BMI), waist circumference, fat mass index (FMI), lean mass index (LMI) and percent body fat at age 18 years.

**Results:**

The final study sample included 1337 participants with anthropometric measurements (52% female) and 958 participants with body composition measurements (53% female). Males who were consistently more active and consistently walked to school over adolescence had lower waist circumference (B = − 2.0, 95% CI: − 3.9 to − 0.2), FMI (B = − 0.8, 95%: CI: − 1.2 to − 0.1) and percent body fat (B = -2.9, 95% CI: − 4.9 to − 0.9) at age 18 years than those who decreased activity and did not walk to school. Consistently-sedentary females had higher waist circumference than those whose sedentary behavior increased over adolescence (B = 5.4, 95% CI: 0.2 to 10.6). Males who reported sleeping 9 h or more per night on schoolnights had significantly lower BMI (B = -1.0, 95% CI: − 1.4 to- 0.5), and percent body fat (B = -1.5, 95%CI − 2.8 to − 0.1) than those who reported sleeping 8 h or less per night.

**Conclusion:**

Patterns of adolescent physical activity, sedentary behavior and sleep are related to young-adult body composition in urban South Africa. These modifiable behaviors may be paths for public health interventions to curb overweight and obesity in many low- or middle-income countries.

**Supplementary Information:**

The online version contains supplementary material available at 10.1186/s12887-020-02451-9.

## Background

Overweight and obesity have become major global health concerns, as both increase the risk of non-communicable diseases (NCD) such as cardiovascular disease, type II diabetes, some cancers, and pre-mature death [1]. The burden of NCD in Africa is similar to the combined burden of communicable, maternal, neonatal and nutritional diseases [2]. South Africa, an upper middle-income country and one of the wealthiest countries on the African continent, also has one of the highest rates of overweight and obesity, affecting 39% of men and 69% of women [3]. As the burdens of overweight and obesity and related NCDs continue to rise in South Africa, there is a need for research on modifiable risk factors in early life that could be integrated into intervention strategies.

Adolescence is marked by rapid physical growth and changes in body composition triggered by the hormonal changes surrounding puberty [4, 5]. Behaviors adopted during adolescence can have both short and long-term effects on health [6]. The health-related behaviors that lead to overweight and obesity in adulthood usually begin or are reinforced during adolescence [7]. Adolescence is also a time of physical activity decline, particularly in females, but most evidence for this comes from high-income countries [8]. Our recent findings suggest that the majority of adolescents in an urban African population, like those from high-income countries, decrease physical activity as they age [9]. Socio-economic status (SES) is closely linked to physical activity, with a previous study in rural South Africa reporting that lower SES was associated with less sedentary time, more walking for transport, and lower moderate-vigorous intensity physical activity, at the maternal, household and community level [10].

Adolescent physical activity [11], sedentary behavior [12] and sleep [13] are modifiable behaviors that have been linked to body composition. However, prospective longitudinal data on these behaviors and how they are associated with body composition in adulthood are rare, and in Africa, non-existent. The benefits of being physically active during adolescence are well documented [14]. Physical activity throughout adolescence has been inversely associated with fat mass and positively associated with lean mass [15]. Further, in the last decade, sedentary behavior has been recognized as a public health concern, independent of physical activity [16]. Adolescents who engage in more than 2 h of sedentary behavior per day have higher risk of unfavorable body composition [17]. Adolescent sleep patterns have also been recognized as relevant for public health concern, with many countries reporting high levels of sleep disturbance among youth [18, 19]. While changes in sleep are a normal part of adolescence, many adolescents accumulate substantial sleep debt, especially during the school week [20]. Inadequate sleep can have consequences for numerous aspects of adolescent health, including risk of overweight and obesity [13].

Previously, we identified categories of distinct sex-specific trends in physical activity, sedentary behavior and sleep, as well as overall physical activity patterns in urban South African adolescents [9]. The associations of these categories and overall patterns with later body composition remain unexplored [9]. The aim of this study was to assess the association of categories of longitudinal physical activity, sedentary behavior and sleep and overall physical activity patterns during adolescence, with body mass index (BMI), waist circumference, fat mass, lean mass and percent body fat at age 18 years in urban South Africa.

## Methods

### Study sample

Participants of this study were part of the Birth-to-Twenty Plus (Bt20+) Cohort, a longitudinal multidisciplinary study of children in Soweto-Johannesburg, South Africa [21]. All singleton children born between April and June 1990, and residing in the area for at least 6 months after birth, were eligible for inclusion [22]. The aim of the original Bt20+ study was to track the growth, health, well-being and educational progress of urban South African children. A total of 3273 children of predominantly low SES were recruited to the study, of whom 78% were Black African and 51% were female [23]. Children were followed up 21 times between birth and age 18 years. Detailed information about the Bt20+ Cohort has been presented elsewhere [21–24]. Due to low study numbers in the other ethnic groups, we restricted our analyses to Black African participants. A flow chart describing the final sample of eligible participants included in the analyses is provided in Supplementary Fig. 1, Additional File 1. Participants or their caregiver gave written informed consent; ethical approval was obtained from the University of the Witwatersrand Committee for Research on Human Subjects (approval ID #M010556).

### Adolescent physical activity, sedentary behavior and sleep trajectories

Detailed methods for the assessment of adolescent behaviors and the use of Latent Class Growth Analysis (LCGA) to determine trajectories, have been described previously [9]. In brief, self-reported physical activity and sedentary behavior were measured annually using a previously validated questionnaire [25]. Adolescents reported frequency and duration spent in informal physical activity (any physical activity outside of school and not part of a sports team or club), organized sports, walking to and from school, and sedentary behavior (time spent before and after school watching TV; reading, drawing and homework; playing a musical instrument; playing video/computer games and internet surfing). Sleep was reported as time the participant went to bed/woke up on schoolnights and the weekend. LCGA is a type of Growth Mixture Modeling (GMM) used to group participants into distinct classes based on common longitudinal trajectories. This method is recommended when a single curve may not adequately describe the variables of interest in a population [26].Using LCGA, participants were grouped into distinct classes based on common longitudinal trajectories of informal activity, organized sports, walking to and from school, sedentary behavior, and schoolnight and weekend sleep. These are depicted graphically in Supplementary Fig. 2, Additional File 2.

### Overall physical activity patterns

Detailed methods for the assessment of overall physical activity patterns have been described previously [9]. In our prior study, individuals who followed similar trajectories of informal activity, organized sports, and walking to and from school synchronously, were categorized into an overall physical activity pattern using group-based multi-trajectory modeling.

### Anthropometric measures

Height was measured without shoes using a stadiometer (Holtain, UK) and recorded to the nearest millimeter. Weight was measured in light clothing using a digital scale (Dismed, USA) and recorded to the nearest 100 g. Both devices were calibrated regularly throughout the study. BMI was calculated as weight (kg) divided by height (m^2^). Waist circumference was measured at the midpoint between the iliac crest and the lowest rib with the participant standing erect and at the end of a normal expiration and recorded to the nearest millimeter. All measurements were taken by trained staff.

### Body composition measures

Whole body fat mass, lean mass and percent body fat at age 18 years were measured using a Hologic QDR 4500A dual-energy x-ray absorptiometry (DXA) scanner and analyzed using Apex software version 4.0.2 (Hologic Inc., Bedford, USA). DXA scans were performed by trained technicians according to standard protocols. The machine was calibrated daily using a phantom spine and coefficient of variation during the course of the study were < 2% for total fat mass, and 1% for lean mass. Fat mass and lean mass are directly associated with height [27]. To account for the association with height, we expressed these variables as fat-mass index (FMI) and lean-mass index (LMI). FMI and LMI were calculated by dividing fat mass and lean mass (in kilograms) by height (in meters) squared [27].

### Selection of covariates

To adjust for potential confounding factors, we selected a series of covariates a priori based on previous findings from this cohort and evidence from the literature [9, 11, 28–34]. These variables were birth order (firstborn/ not firstborn), SES asset quintile at child age 7/8 years (a time point designed to precede the pubertal growth spurt at which comprehensive measures were obtained), maternal schooling (matriculated/did not matriculate from secondary school) at child age 7/8 years, maternal marital/union status (in union/not in union) at child age 7/8 years and childhood BMI z score (BMIZ) at child age 7/8 (or 9, if not available at age 7/8) years. Childhood BMIZ was determined by converting BMI values to BMI z-scores using the WHO reference [35]. If the participant had a BMI measurement at age 7/8 years, that data point was used to calculate childhood BMIZ. If the participant did not have a BMI measurement at age 7/8 years but had one at age 9 years, then the measurement at age 9 years was used to calculate childhood BMIZ. This allowed us to calculate childhood BMIZ for 65% of participants. Co-linearity between these variables was examined using variance inflation factors and was not notable. Additional details on the selection and measurement of covariates can be found elsewhere [9].

### Inclusion and exclusion

To be included in the present analyses, participants needed to have been included in the physical activity, sedentary behavior and sleep trajectories (which requires at least two data points between 12 and 18 years of age), and have either anthropometric or body composition data at age 18 years.

### Statistical analyses

We compared the BMI, waist circumference, FMI, LMI and percent body fat between the physical activity, sedentary behaviour and sleep groups for males and females using analysis of variance (ANOVA). We used general linear models to investigate the associations between categories of longitudinal adolescent physical activity, sedentary behavior and sleep and overall activity patterns, with BMI, waist circumference, FMI, LMI and percent body fat at age 18 years. We stratified all analyses by sex due to previously observed sex-differences in patterns of adolescent physical activity, sedentary behavior and sleep, and overweight and obesity in this population [9, 36]. We adjusted models for birth order, maternal schooling at child age 7/8 years, maternal marital/union status at child age 7/8 years, SES quintile at child age 7/8 years and childhood BMIZ at ages 7/8 or 9 years. For analyses that included overall physical activity patterns, we also adjusted for adolescent sedentary behavior and schoolnight sleep trajectories. We conducted all analyses in Stata version 14 and set significance at *p* < 0.05.

## Results

The final study sample included 1337 participants with anthropometric measurements (52% female), and 958 participants with DXA-derived body composition measurements (53% female). Black-African participants excluded from the analyses were similar to those included in the analyses, and participants who had both anthropometric and body composition data at age 18 years were similar to participants who had only anthropometric data (Supplementary Table 1, Additional File 3). Characteristics of the study sample are provided in Table 1.

**Table 1:** Characteristics of participants by sex, Bt20+

	Males	Females
N	638	699
Childhood characteristics		
Birth order, %		
First born	38	41
BMIZ at age 7/8 or 9 y^a^	0.0 (0.9)	0.0 (1.0)
Maternal characteristics		
Schooling at child age 7/8 y, %		
Matriculated	29	31
Household characteristics		
SES asset quintile at child age 7/8 y, %		
1 (lowest)	16	22
2	21	17
3	30	29
4	23	19
5 (highest)	10	13
Adolescent trajectories		
**informal activity, %**		
Decreasing over time	93	95
Increasing over time	7	5
**Organized sports participation, %**		
Males		
Consistently low	82	
Decreasing from adequate to low	11	
Increasing from low to adequate	7	
Females		
No sports participation		89
Some sports participation		11
**Walking to and from school, %**		
Consistently 150 min/week	83	75
Consistently 300 min/week	17	25
**Overall physical activity, %**		
Decreasing activity over time and do not walk to school	22	31
Decreasing activity over time but consistently walk to school	50	54
Consistently more active and consistently walk to school	28	15
**Sedentary behavior, %**		
Males		
Consistently low	78	
Consistently high	12	
Increasing from low to high	10	
Females		
Initially low but increasing		92
Consistently high		8
**Schoolnight sleep, %**		
≤ 8 h/night	35	65
≥ 9 h/night	65	35
Young-adult characteristics (18 y)		
Height^a^, cm	170.7 (6.5)	159.6 (6.1)
BMI^a^, kg/m2	20.3 (3.2)	23.2 (4.6)
Waist-circumference^a^, cm	72.0 (7.2)	75.6 (12.9)
Fat mass index^a,b^, kg/m^2^	2.5 (1.5)	7.3 (3.0)
Lean mass index^a,b^, kg/m^2^	15.0 (1.5)	13.1 (1.6)
Body fat^a,b^, %	13.2 (5.5)	33.6 (6.7)

^a^ Expressed as mean (standard deviation)

^b^ data collected from only 958 participants (449 males and 509 females)

### Physical activity- males

93% of males decreased informal activity over adolescence. In unadjusted models, males who increased informal activity over adolescence had significantly lower BMI, FMI and percent body fat than those whose informal activity decreased (Table 2). Using the decreasing informal activity as the reference group in the regression model, these differences were attenuated following covariate adjustment (Table 3).

**Table 2:** Mean anthropometric and body composition measures at 18y by adolescent movement behavior trajectories, Bt20+

N	BMI (kg/m^2^)	Waist circumference (cm)	Fat Mass Index^a^ (kg/m^2^)	Lean Mass^b^ Index^c^ (kg/m^2^)	Body fat (%)	BMI (kg/m^2^)	Waist circumference (cm)	Fat Mass Index (kg/m^2^)	Lean Mass Index (kg/m^2^)	Body fat (%)
	Males					Females				
	638	638	449	449	449	699	699	509	509	509
	Mean (SD)	Mean (SD)	Mean (SD)	Mean (SD)	Mean (SD)	Mean (SD)	Mean (SD)	Mean (SD)	Mean (SD)	Mean (SD)
**Informal activity**										
Decreasing over time	20.4 (3.1)	72.1 (7.3)	2.5 (1.6)	15.7 (1.5)	13.4 (5.6)	23.1 (4.6)	75.4 (11.0)	7.2 (3.0)	13.1 (1.6)	33.5 (7.0)
Increasing over time	19.4 (2.1)	69.9 (5.1)	1.9 (0.7)	14.7 (1.0)	11.1 (3.4)	23.9 (4.3)	80.1 (31.1)	8.4 (2.8)	13.8 (1.5)	35.8 (6.5)
P-value	0.04	0.05	0.04	0.21	0.03	0.3	0.03	0.08	0.03	0.12
**Organized sports participation**										
Males										
Consistently low	30.4 (3.2)	72.1 (7.5)	2.5 (1.6)	15.0 (1.5)	13.4 (5.6)					
Decreasing from adequate to low	19.8 (2.3)	71.5 (6.4)	2.3 (1.7)	14.7 (1.1)	12.6 (6.1)					
Increasing from low to adequate	20.1 (1.9)	71.2 (5.0)	2.2 (0.9)	15.3 (1.4)	12.1 (4.0)					
P-value	0.4	0.6	0.3	0.3	0.3					
Females										
None						23.3 (4.7)	76.0 (13.2)	7.4 (3.01)	13.1 (1.7)	33.9 (6.9)
Consistently some						22.3 (3.4)	72.8 (9.1)	6.5 (2.7)	13.1 (1.2)	31.1 (7.5)
P-value						0.1	0.04	0.04	0.9	0.005
**Walking to and from school**										
Consistently 150 min/week	20.4 (3.1)	72.2 (7.4)	2.6 (1.6)	15.1(1.5)	13.4 (5.7)	23.2 (4.8)	75.8 (13.6)	7.4 (3.2)	13.1 (1.7)	33.7 (7.3)
Consistently 300 min/week	19.9 (2.6)	71.0 (6.1)	2.2 (1.0)	14.9 (1.5)	12.1 (4.1)	23.0 (3.7)	75.2 (10.6)	7.1 (2.5)	13.2 (1.4)	33.2 (6.2)
P-value	0.2	0.1	0.07	0.3	0.07	0.7	0.6	0.5	0.4	0.4
**Overall physical activity** ^**d**^										
Decreasing activity over time and do not walk to school	20.9 (3.6)	72.3 (7.4)	3.0 (2.2)	15.1 (1.4)	14.9 (7.4)	23.6 (5.2)	75.6 (11.7)	7.7 (3.3)	13.1 (1.7)	34.7 (7.2)
Decreasing activity over time but consistently walk to school	20.4 (3.1)	72.3 (7.3)	2.5 (1.3)	15.0 (1.6)	13.2 (4.9)	23.0 (4.3)	75.7 (11.0)	7.2 (3.0)	13.1 (1.6)	33.4 (6.8)
Consistently more active and consistently walk to school	19.3 (2.0)	70.1 (6.0)	2.1 (1.1)	15.1 (1.2)	11.7 (4.3)	22.8 (3.9)	75.5 (20.0)	6.8 (2.7)	13.2 (1.4)	32.1 (7.1)
P-value	0.001	0.03	0.0002	0.7	0.0001	0.2	0.9	0.1	0.8	0.03
**Sedentary behavior**										
Males										
Consistently low	20.1 (2.6)	71.5 (6.5)	2.5 (1.5)	15.0 (1.5)	13.2 (5.5)					
Consistently high	20.7 (4.4)	72.6 (9.2)	2.1 (0.9)	14.7 (1.4)	12.0 (4.4)					
Increasing from low to high	21.2 (3.8)	74.6 (8.8)	2.8 (1.8)	15.3 (1.4)	14.1 (6.3)					
P-value	0.004	0.001	0.2	0.3	0.3					
Females										
Initially low but increasing						23.1 (4.5)	75.2 (10.9)	7.3 (3.0)	13.1 (1.6)	33.6 (6.9)
Consistently high						24.3 (5.0)	81.3 (26.7)	7.8 (3.6)	13.7 (1.7)	33.9 (8.4)
P-value						0.05	0.0008	0.4	0.04	0.8
**Schoolnight sleep**										
≤ 8 h/night	20.9 (3.7)	73.3 (8.5)	2.7 (1.7)	15.1 (1.6)	13.9 (5.9)	23.3 (4.6)	75.7 (14.0)	7.4 (2.9)	13.2 (1.6)	33.8 (6.9)
≥ 9 h/night	20.0 (2.5)	71.2 (6.2)	2.4 (1.4)	15.0 (1.4)	12.9 (5.3)	22.8 (4.5)	75.4 (10.6)	7.1 (3.2)	13.0 (1.7)	33.1 (7.2)
P-value	0.0001	0.0007	0.06	0.3	0.06	0.1	0.8	0.3	0.2	0.3

^a^ Fat mass index, calculated by dividing fat mass (in kg) by height (in meters) squared

^b^ Lean mass does not include bone mineral content

^c^ Lean mass index, calculated by dividing lean mass (in kg) by height (in meters) squared

^d^ Multi-trajectory groups made up of informal activity, organized sports and walking to and from school trajectories, representing overall physical activity pattern

*P*-values represent comparisons between groups for males and females, using ANOVA

**Table 3:** Regression coefficients (B) and 95% CI for anthropometric and body composition measures at 18y, adjusted, Bt20+

	BMI (kg/m2)		Waist circumference (cm)		Fat mass index ^a^ (kg/m2)		Lean mass ^b^ index ^c^ (kg/m2)		Body fat (%)	
**Males**										
N	B (95% CI)	*P*-value	B (95% CI)	*P*-value	B (95% CI)	*P*-value	B (95% CI)	*P*-value	B (95% CI)	*P*-value
**Informal activity** ^**d**^										
Decreasing over time	ref	0.7	ref	0.9	ref	0.5	ref	0.7	ref	0.4
Increasing over time	−0.2(−1.1–0.68)		0.0 (−2.4–2.4)		− 0.2 (− 0.8–0.4)		0.1 (− 0.5–0.7)		−0.9 (− 3.3–1.4)	
**Organized Sports Participation** ^**d**^										
Consistently low	ref	0.4	ref	0.2	ref	0.5	ref	0.5	ref	0.4
Decreasing from adequate to low	0.02 (−0.9–0.8)		0.8 (−1.-3.0)	0.5	0.1 (−0.5–0.8)		− 0.2 (− 0.8–0.3)		0.4 (−2.0–2.8)	
Increasing from low to adequate	−0.2 (− 1.1–0.7)		−1.6 (− 3.7–0.4)	0.1	− 0.3 (− 0.9–0.3)		0.1 (− 0.4–0.7)		−1.4 (− 3.6–0.8)	
**Walking to and from school** ^**d**^										
Consistently 150 min/week	ref	0.7	ref	0.8	ref	0.4	ref	0.8	ref	0.3
Consistently 300 min/week	− 0.1 (−0.7–0.5)		− 0.2 (−1.9–1.5)		− 0.2 (− 0.7–0.3)		0.0 (− 0.4–0.5)		−0.9 (− 2.7–0.9)	
**Overall physical activity** ^**e**^										
Model 1 ^d^										
Decreasing activity over time and do not walk to school	ref	0.02	ref	0.01	ref	0.01	ref	0.5	ref	0.004
Decreasing activity over time but consistently walk to school	−0.8 (−1.4--0.2)		−2.1 (− 3.7--0.4)		− 0.7 (− 1.1–0.2)		−0.2 (− 0.6–0.2)		−2.2 (− 3.8--0.6)	
Consistently more active and consistently walk to school	− 0.8 (− 1.5--0.2)		− 2.7 (−4.5−− 0.8)		-0.8 (− 1.3--0.3)		0.0 (− 0.5–0.4)		−3.2 (−5.1--1.3)	
Model 2 ^f^										
Decreasing activity over time and do not walk to school	ref	0.1	ref	0.1	ref	0.02	ref	0.6	ref	0.008
Decreasing activity over time but consistently walk to school	−0.6 (−1.2–0.1)		−1.4 (−3.1–0.3)		− 0.56(− 1.0−− 0.1)		-0.1 (− 0.5–0.3)		−1.9 (− 1.7--0.2)	
Consistently more active and consistently walk to school	− 0.6 (− 1.3–0.0)		−2.0 (− 3.9--0.2)		−0.8 (− 1.2--0.1)		0.1 (− 0.4–0.6)		−2.9 (−4.9--0.9)	
**Sedentary behavior** ^**d**^										
Consistently low	ref	0.1	ref	0.2	ref	0.2	ref	0.4	ref	0.2
Consistently high	0.6 (−1.3–1.4)		0.1 (−2.1–2.2)		−0.7 (− 1.5–0.2)		−0.2 (− 0.9–0.6)		−2.7 (−5.8–0.5)	
Increasing from low to high	0.5 (− 0.2–1.2)		1.8 (− 0.1–3.8)		0.3 (− 0.2–0.8)		0.3 (− 0.2–0.8)		0.6 (− 1.3–2.5)	
**Schoolnight sleep** ^**d**^										
≤ 8 h/night	ref		ref		ref		ref		ref	
≥ 9 h/night	−1.0 (− 1.4--0.5)	< 0.0001	− 2.3 (− 3.7--1.1)	< 0.0001	− 0.4 (− 0.8--0.1)	0.02	0.1 (− 0.2–0.5)	0.5	−1.5 (− 2.8--0.1)	0.04
**Females**										
N	B (95% CI)	*P*-value	B (95% CI)	P-value	B (95% CI)	*P*-value	B (95% CI)	*P*-value	B (95% CI)	*P*-value
**Informal activity** ^**d**^										
Decreasing over time	ref	0.2	ref	0.02	ref	0.2	ref	0.2	ref	0.2
Increasing over time	0.9 (−0.4–2.1)		6.0 (0.9–11.1)		0.8 (− 0.5–2.0)		0.4 (− 0.2–1.1)		2.1 (−1.1–5.3)	
**Organized sports participation** ^**d**^										
None	ref	0.8	ref	0.6	ref	0.9	ref	0.05	ref	0.6
Consistently some	0.4 (−0.6–1.4)		−1.4 (−5.0–2.7)		0.1 (− 0.9 1.0)		0.5 (0.0–1.0)		− 0.7 (−3.0–1.67	
**Walking to and from school** ^**d**^										
Consistently 150 min/week	ref	0.3	ref	0.8	ref	0.7	ref	0.02	ref	0.9
Consistently 300 min/week	0.4 (−0.3–1.1)		0.4 (−3.4–2.6)		0.1 (−0.5–0.8)		0.4 (0.1–0.7)		0.1 (−1.5–1.7)	
**Overall physical activity** ^**e**^										
Model 1 ^d^										
Decreasing activity over time and do not walk to school	ref	0.2	ref	0.09	ref	0.5	ref	0.2	ref	0.6
Decreasing activity over time but consistently walk to school	0.5 (−0.3–1.3)		2.6 (−0.6–5.8)		0.5 (−0.3–1.2)		0.3 (− 0.1–0.7)		0.9 (−1.0–2.7)	
Consistently more active and consistently walk to school	0.9 (−0.1–1.9)		4.6 (0.5–8.6)		0.4 (−0.6–1.3)		0.4 (− 0.1–0.9)		0.1 (−2.3–2.6)	
Model 2 ^f^										
Decreasing activity over time and do not walk to school	ref	0.2	ref	0.07	ref	0.5	ref	0.2	ref	0.6
Decreasing activity over time but consistently walk to school	0.5 (−0.3–1.4)		2.9 (− 0.4–6.1)		0.4 (− 0.3–1.2)		0.3 (− 0.1–0.7)		0.8 (−1.1–2.7)	
Consistently more active and consistently walk to school	0.9 (− 0.1–2.0)		4.8 (0.7–9.0)		0.4 (− 0.6–1.3)		0.5 (0.0–0.1)		0.1 (−2.3–2.6)	
**Sedentary behavior** ^**d**^										
Initially low but increasing	ref	0.08	ref	0.04	ref	0.1	ref	0.7	ref	0.06
Consistently high	−1.1 (−2.4–0.1)	0.08	5.4 (0.2–10.6)		−1.2 (−2.6–0.2)		−0.2 (−0.9–0.6)		−3.5 (−7.0–0.1)	
**Schoolnight sleep** ^**d**^										
≤ 8 h/night	ref	0.8	ref	0.6	ref	0.3	ref	0.7	ref	0.7
≥ 9 h/night	0.1 (−0.6–0.8)		0.6 (− 2.2–3.3)		0.3 (− 0.3–0.9)		−0.1 (− 0.4–0.2)		0.3 (− 1.2–1.8)	

^a^ Fat mass index, calculated by dividing fat mass (in kg) by height (in meters) squared

^b^ Lean mass does not include bone mineral content

^c^ Lean mass index, calculated by dividing lean mass (in kg) by height (in meters) squared

^d^ Adjusted for childhood BMIZ at age 7/ 8 or 9 years, birth order, maternal schooling, maternal marital status and SES asset quintile at child age 7/8 years

^e^ Multi-trajectory groups made up of informal activity, organized sports and walking to and from school trajectories representing overall physical activity pattern

^f^ Adjusted for childhood BMIZ at age 7/ 8 or 9 years, birth order, maternal schooling, maternal marital status and SES asset quintile at child age 7/8 years, and sedentary behavior and sleep trajectories

82% of males had consistently low sports participation over adolescence (less than the median of 186 mins/wk., Supplementary Fig. 2), while 11% decreased from adequate to low, and 7% increased from low to adequate. In unadjusted and adjusted (using consistently low sports participation as the reference group) models, there were no associations between trends in organized sports participation and any anthropometric or body composition outcome at age 18 years.

83% of males walked approximately 150 min per week to and from school over adolescence, while the remainder walked approximately 300 min per week. In both unadjusted and adjusted (using walking consistently 150 mins/wk. as the reference group) models, there were no associations between the amount of time spent walking to and from school during adolescence and any anthropometric or body composition outcome at age 18 years in males.

### Physical activity- females

95% of females decreased informal activity over adolescence. In both unadjusted and adjusted (using decreased informal activity as the reference group) models, females who increased informal activity over adolescence had significantly higher waist circumference than females whose informal activity decreased. LMI was also higher among females whose informal activity increased, but only in the unadjusted models.

89% of adolescent females did not participate in organized sports. In unadjusted models, females who consistently participated in organized sports over adolescence had significantly lower waist circumference, FMI and percent body fat than those who did not participate in organized sports. These differences were attenuated following covariate adjustment (using consistently low sports participation as the reference group in the regression model).

75% of females walked approximately 150 min per week to and from school over adolescence, while the remainder walked approximately 300 min per week. In unadjusted models, there were no differences in any anthropometric or body composition outcome at age 18 years between these groups. However, using walking approximately 150 min per week as the reference group and adjusting for covariates, females who walked approximately 300 min per week to and from school over adolescence had significantly higher LMI (B = 0.4, 95% CI: 0.1 to 0.7).

### Overall physical activity

22% of males decreased activity and did not walk to school over adolescence, 50% decreased activity and walked to school, and 28% were consistently more active and walked to school. In unadjusted models, trends in male overall adolescent physical activity were associated with BMI, waist circumference, FMI and percent body fat at age 18 years. After adjusting for childhood and maternal covariates (model 1), when compared to the reference (males who decreased physical activity and did not walk to school), the other two groups had lower BMI, waist circumference, FMI, and percent body fat at age 18 years. After also adjusting for adolescent sedentary behavior and sleep trajectories (model 2), when compared to the same reference group, males who decreased activity and walked to school over adolescence had significantly lower FMI (B = -0.6, 95% CI: − 1.0 to − 0.1) and percent body fat (B = -1.9, 95% CI: − 1.7 to − 0.2), and males who were consistently more active and walked to school over adolescence had significantly lower waist circumference (B = − 2.0, 95% CI: − 3.9 to − 0.2), FMI (B = − 0.8, 95%: CI: − 1.2 to − 0.1) and percent body fat (B = -2.9, 95% CI: − 4.9 to − 0.9) at age 18 years.

31% of females decreased activity and did not walk to school over adolescence, 54% decreased activity and walked to school, and 15% were consistently more active and walked to school. In unadjusted models, trends in female overall adolescent physical activity were associated with percent body fat at age 18 years. However, after adjusting for covariates, there were no significant differences in anthropometric or body composition outcomes at age 18 years between females who decreased activity but walked to school over adolescence and those who decreased activity and did not walk to school. In models adjusted for childhood and maternal covariates (model 1), and models also adjusted for sedentary behavior and schoolnight sleep trajectories (model 2), females who were consistently more active and consistently walked to school over adolescence had significantly higher waist circumference at age 18 years than females who decreased activity and did not walk to school over adolescence, the reference group (B = 4.6, 95% CI: 0.5–8.6) and (B = 4.8, 95% CI: 0.7–9.0).

### Sedentary behavior

78% of males engaged in consistently low amounts of sedentary behavior over adolescence, while 10% engaged in consistently high amounts, and 12% increased from low to high amounts. In unadjusted models, trends in male sedentary behavior over adolescence were associated with BMI and waist circumference at age 18 years with males whose sedentary behavior increased during adolescence having higher BMI and waist circumference. These associations were attenuated following covariate adjustment, with consistently low sedentary behavior used as the reference group.

In females, 92% engaged in initially low but increasing amounts of sedentary behavior and 8% engaged in consistently high amounts (> median of 2000 mins/wk). In unadjusted models, trends in female sedentary behavior were associated with waist circumference and LMI. After adjusting for covariates, females who engaged in consistently high amounts of sedentary behavior over adolescence had significantly higher waist circumference than those who engaged in initially low but increasing amounts, the reference group (B = 5.4, 95% CI: 0.2 to 10.6).

### Sleep

35% of males and 65% of females reported sleeping for 8 h or less on schoolnights. In unadjusted models, males who reported sleeping 9 h or more per night on schoolnights over adolescence had significantly lower BMI and waist circumference at age 18 years than those who reported sleeping 8 h or less per night. After adjusting for covariates, males who reported sleeping 9 h or more per night on schoolnights had significantly lower BMI (B = -1.0, 95% CI: − 1.4 to- 0.5), waist circumference (B = -2.3, 95% CI − 3.7 to − 1.1), FMI (B = -0.4, 95% CI: − 0.8 to − 0.1) and percent body fat (B = -1.5, 95%CI − 2.8 to − 0.1) than those who reported sleeping 8 h or less per night, the reference group. In both unadjusted and adjusted models, there were no associations between schoolnight sleep in adolescent females and any anthropometric or body composition outcomes at age 18 years.

## Discussion

This study related categories of longitudinal adolescent physical activity, sedentary behavior and sleep, as well as overall adolescent physical activity patterns, to young adult body composition in an urban South African birth cohort. Given the high burden of overweight and obesity in South Africa, there is interest in lifestyle behaviors that can be modified earlier in life. There were three main findings in this study. First, after controlling for covariates, being consistently more active throughout adolescence was associated with more favorable anthropometric measures and body composition at age 18 years in urban South African males. Second, engaging in consistently high amounts of sedentary behavior throughout adolescence was associated with higher waist circumference at age 18 years in females. Finally, sleeping 9 h or more per night on schoolnights was associated with a more favorable anthropometric measures and body composition at age 18 years in urban South African males.

Adolescence is a period of rapid growth and maturation characterized by significant changes in body composition [37]. While genetics, nutrition and hormones are the primary drivers of these changes, physical activity is an important ancillary determinant of body composition and may play an important role in increasing muscle mass and preventing overweight and obesity [38].

An age-related decline in physical activity throughout adolescence has been well established in high-income countries [8]. In our study population, the large majority of both males (72%) and females (85%) decreased their reported levels of physical activity over adolescence [9]. This decline is potentially a risk factor for adult overweight or obesity [39–41]. Until now, prospective evidence from Africa has been lacking. Our study found that Black-African males who were consistently more active over adolescence had significantly lower waist circumference, FMI and percent body fat in young-adulthood compared to Black-African males who decreased overall activity over adolescence. These results are consistent with findings from the Iowa Bone Development Study cohort, in which participants who were physically active as children but decreased activity with age were more likely to become obese in young-adulthood than participants who were consistently active [39]. In contrast, we found that being consistently more active during adolescence in Black-African females was not associated with more favorable body composition in young-adulthood, and to be associated with higher waist circumference, a proxy for abdominal obesity.

Due to hormonal changes, adolescence is a period of fat mass accretion in females [37]. Fat mass accretion, combined with the lower amount of, and likely less intense, physical activity may explain the differences in our finding among males and females. Qualitative research has discovered that particularly for girls, physical activity declines during the secondary school years as girls are more conscious of their appearance during puberty and cultural norms tend to favor fuller body shapes [39, 40]. The association between higher physical activity and higher waist circumference among females may be due to other factors, including diet, over-riding the influence of physical activity on abdominal obesity.

In a study from another low-or middle-income country, the 1993 Pelotas (Brazil) Birth Cohort Study, consistent moderate-to-vigorous intensity physical activity (MVPA) over adolescence was associated with higher lean mass at age 18 years in males and females [15]. In the present study, we considered organized sports a proxy for MVPA. In contrast to the Brazilian cohort, we found that trends in participation in organized sports were not associated with lean mass or any other measure of body composition at age 18 years, in either males or females. The inconsistency in findings between our study and the Brazilian cohort might be explained by low levels of activity in the South African cohort, as 82% of males and all females failed to meet the WHO recommendation of 60 min of MVPA per day, as compared to 37 and 65% in the Pelotas cohort [42].

Informal activity, also known as active play, may make a significant contribution to an adolescent’s daily energy expenditure [42, 43]. While unstructured playtime is typically not very intense, the long duration of activity may lead to benefits [44]. In our study, we found no association between trends in informal activity over adolescence and any anthropometric or body composition outcome at age 18 years in males. However, we found females who increased informal activity over adolescence had significantly higher waist circumference at age 18 years than females who decreased informal activity over adolescence. The duration of male informal activity was likely not high enough to compensate for the low intensity. The association between trends in informal activity and waist circumference in females was not as hypothesized and may have been influenced by unmeasured variables.

Active transportation to school has been identified as an important source of physical activity in adolescents, but there is conflicting evidence regarding the association between active transportation to school and body composition [45, 46]. In our study, walking to and from school was a consistent source of physical activity over adolescence. While trends in walking to and from school over adolescence were not associated with anthropometric measures or body composition at age 18 years in males, we found that females who consistently spent more time walking to and from school over adolescence had significantly higher LMI at age 18 years than females who spent less time walking. This association, however, was only significant after adjusting for childhood BMIZ, indicating the possibility of negative confounding from an unmeasured variable. In the 1993 Pelotas Birth Cohort, active commuting to school throughout adolescence was associated with lower levels of central fatness at age 18 years in males [47]. It is possible that the intensity of walking to and from school was not high enough to have an impact on adiposity in our South African population. The Pelotas study included commuting via bicycle in their measure of active commuting, while we only included walking due to low prevalence of reported cycling.

Sedentary behavior, independent of physical activity, is a risk factor for overweight and obesity [16]. Sedentary behaviors, such as TV viewing, in childhood and adolescence have been prospectively linked to overweight and other adverse health outcomes in adulthood [48]. In our study, females who engaged in consistently high amounts of sedentary behavior throughout adolescence had significantly higher waist circumference at age 18 years than females who engaged in initially low but increasing amounts of sedentary behavior, but there was no association between trends in sedentary behavior and anthropometric measures and body composition at age 18 years in males. In an Australian birth cohort, males with lower screen time in childhood had lower percent body fat at age 20 years than males with consistently high screen time, and females with initially and consistently lower screen time had lower percent fat at age 20 years than females with consistently high screen time [12]. The Australian study focused on TV viewing, whereas we examined total sedentary behavior and individual sedentary behaviors that have been shown to have varying impacts on different health outcomes [49].

Reduced sleep is an independent risk factor for excess weight [50, 51]. Proposed mechanisms behind this relationship include metabolic and endocrine alterations, increased appetite leading to higher caloric consumption, increased systemic inflammation and decreased physical activity related to daytime sleepiness [52–54]. Most of the evidence on sleep duration and excess weight is cross-sectional, comes from high-income countries, or did not follow up into adulthood [13]. We found that males who reported consistently getting more sleep per night on schoolnights had significantly lower BMI, waist circumference, fat mass index and percent body fat at age 18 years than males who reported sleeping fewer hours per night. Trends in sleep duration over adolescence, however, were not associated with anthropometric measures or body composition at age 18 years in females. In support of our findings, Knutson (2005) found the association between short sleep duration and obesity to only exist in males [55]. The sex difference in this association may be attributed to different physiological mechanisms and hormones in males and females [55]. In contrast to our sex-specific findings, in the Pelotas Birth Cohort Study, females who increased from inadequate (< 8 h per day) to adequate (≥8 h per day) sleep duration over adolescence had higher BMI, fat mass index and fat-free mass index at age 18 years than those who consistently got adequate sleep [56]. Differences in adolescent sleep trends between the South African and Brazilian cohorts and differences in how sleep duration was categorized may explain the differences in findings.

### Strengths and limitations

The main strength of our study was the use of longitudinal data with follow-up into late adolescence. We used longitudinal data from Africa, which is particularly scarce. Additionally, the use of detailed body composition data obtained via DXA allowed us to distinguish between fat mass and lean mass, providing a more accurate predictor of overweight and obesity than can be obtained from the use of BMI alone. The main limitation of our study was the use of self-reported physical activity, sedentary behavior and sleep data. Additionally, the LCGA and multi-trajectory modeling used previously to generate adolescent trajectories and overall physical activity patterns come with limitations, which have been discussed in detail elsewhere [9]. In brief, the validity of LCGA may be challenged as model fit statistics may not sufficiently differentiate between a model with many latent classes, and a single class model with non-normal outcomes. Also, this study cannot address the potential impact of dietary patterns on the relationship between movement behaviors and adiposity. Regardless of its limitations, this study contributes to the scarcity of prospective data on adolescent health behaviors and adult health in Africa.

## Conclusion

Being consistently physically active and getting more sleep per night throughout adolescence was associated with more favorable body composition at age 18 years in urban South African males. Being more sedentary over adolescence was associated with less favorable anthropometric measurements in urban South African females. Adolescent physical activity, sedentary behavior and sleep are modifiable behaviors that may be contributing to the prevalence of overweight and obesity in urban South African adults.

## Supplementary Information

**Additional file 1: Supplementary Fig. 1**. Flow chart depicting the final sample of eligible participants included in the analysis, Bt20+.

**Additional file 2: Supplementary Fig. 2**. Trajectories of physical activity and sedentary behavior from Latent Class Growth Analysis, Bt20+.

**Additional file 3: Supplementary Table 1**. Comparison of characteristics of the study sample by data available and sex, Bt20+.

## Data Availability

The datasets used and/or analyzed during the current study are available from Professor Shane Norris (shane.norris@wits.ac.za) on reasonable request.

## Abbreviations

BMI: Body Mass Index
BMIZ: Body Mass Index Z-score
Bt20 +: Birth to Twenty Plus Cohort
DXA: Dual-energy X-ray Absorptiometry
FMI: Fat Mass Index
LCGA: Latent Class Growth Analysis
LMI: Lean Mass Index
MVPA: Moderate-to-Vigorous Intensity Physical Activity
NCD: Non-Communicable Disease
WHO: World Health Organization
