# Supplementary material for: Adolescent physical activity, sedentary behavior and sleep in relation to body composition at age 18 years in urban South Africa, Birth-to-Twenty+ Cohort
Source: BMC Pediatr. 2021 Jan 11;21:30. doi: 10.1186/s12887-020-02451-9 (PMC7798220; doi:10.1186/s12887-020-02451-9)
Supplement: Supplementary file 1 — Additional file 1: Supplementary Fig. 1. Flow chart depicting the final sample of eligible participants included in the analysis, Bt20+. [file 12887_2020_2451_MOESM1_ESM.pdf]

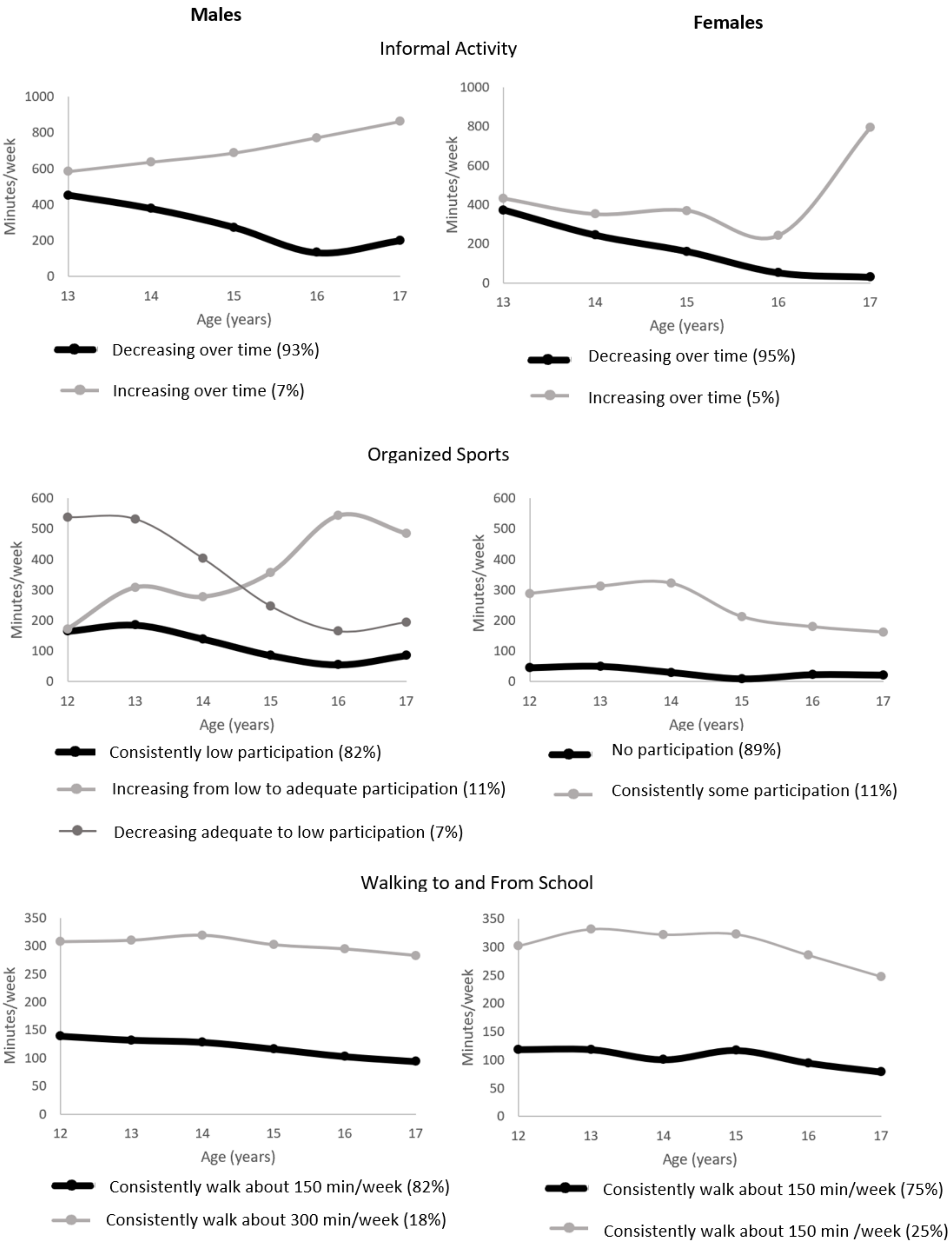
Males
Females
Informal Activity
Minutes/week
Age (years)
Decreasing over time (93%)
Increasing over time (7%)
Decreasing over time (95%)
Increasing over time (5%)
Organized Sports
Consistently low participation (82%)
Increasing from low to adequate participation (11%)
Decreasing adequate to low participation (7%)
No participation (89%)
Consistently some participation (11%)
Walking to and From School
Consistently walk about 150 min/week (82%)
Consistently walk about 300 min/week (18%)
Consistently walk about 150 min/week (75%)
Consistently walk about 150 min /week (25%)

Sedentary Behavior

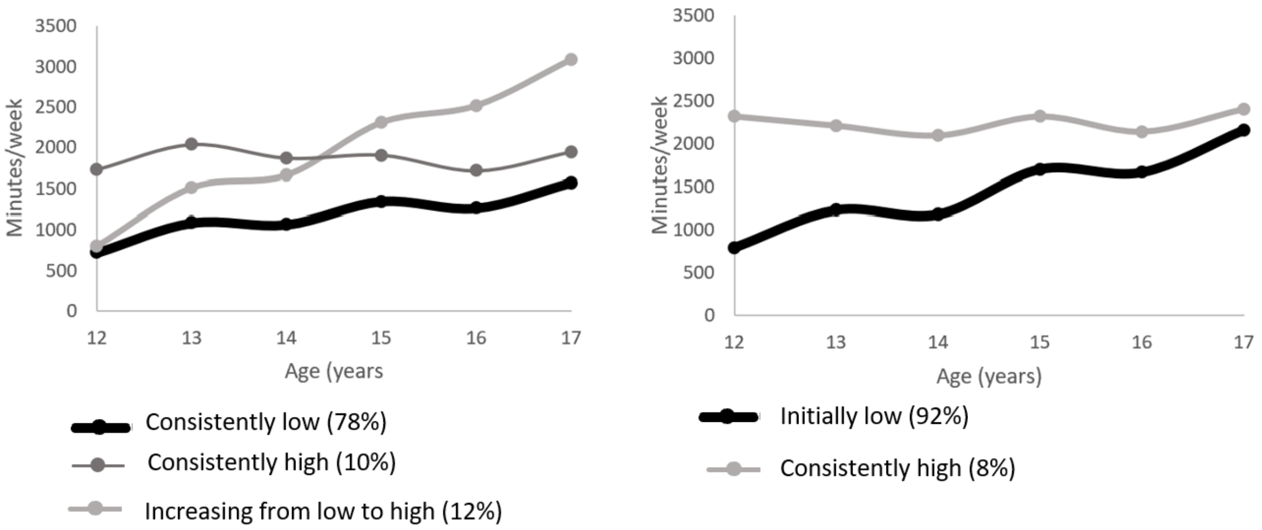

[a] Figure has been adapted from previously published work and is intended to provide necessary background information for the present study [8] The use of this figure is goverend by the copyright release in the original manuscript.

Supplementary Figure 1. Trajectories of physical activity and sedentary behavior from Latent Class Growth Analysis, Bt20+
