# Supplementary material for: Adolescent physical activity, sedentary behavior and sleep in relation to body composition at age 18 years in urban South Africa, Birth-to-Twenty+ Cohort
Source: BMC Pediatr. 2021 Jan 11;21:30. doi: 10.1186/s12887-020-02451-9 (PMC7798220; doi:10.1186/s12887-020-02451-9)
Supplement: Supplementary file 2 — Additional file 2: Supplementary Fig. 2. Trajectories of physical activity and sedentary behavior from Latent Class Growth Analysis, Bt20+. [file 12887_2020_2451_MOESM2_ESM.pdf]

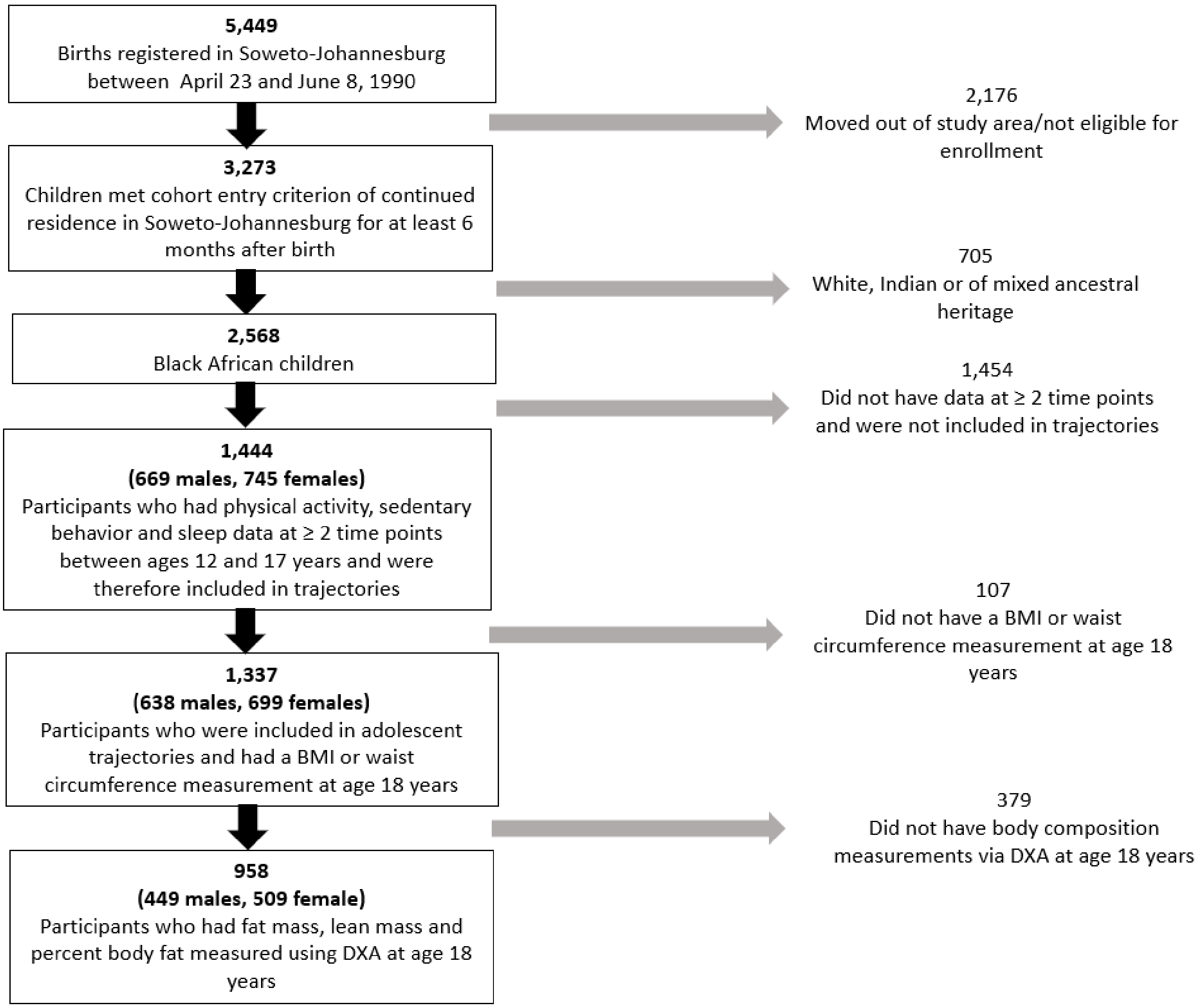

Supplementary Figure 2. Flow chart depicting the final sample of eligible participants included in the analysis, Bt20+
