# Supplementary material for: Adolescent physical activity, sedentary behavior and sleep in relation to body composition at age 18 years in urban South Africa, Birth-to-Twenty+ Cohort
Source: BMC Pediatr. 2021 Jan 11;21:30. doi: 10.1186/s12887-020-02451-9 (PMC7798220; doi:10.1186/s12887-020-02451-9)
Supplement: Supplementary file 3 — Additional file 3: Supplementary Table 1. Comparison of characteristics of the study sample by data available and sex, Bt20+. [file 12887_2020_2451_MOESM3_ESM.docx]

Supplementary table 1. Comparison of characteristics of the study sample by data available and sex, Bt20+

|  | **Males** | | | | | **Females** | | | | |
| --- | --- | --- | --- | --- | --- | --- | --- | --- | --- | --- |
|  | Black-Africans Excluded ^a^ | P-value ^b^ | Anthro Only ^c^ | Anthro + DXA ^d^ | P-value | Black-Africans Excluded | P-value | Anthro Only | Anthro + DXA | P-value |
| N | 608 |  | 189 | 449 |  | 623 |  | 190 | 509 |  |
| Childhood characteristics |  |  |  |  |  |  |  |  |  |  |
| Birth order, % |  |  |  |  |  |  |  |  |  |  |
| First born | 33 | 0.04 | 41 | 37 | 0.4 | 32 | 0.001 | 44 | 40 | 0.8 |
| BMIZ at age 7/8 or 9 y | -- | -- | 0.1 (0.9) | 0.0 (0.9) | 0.1 | -- | -- | 0.0 (0.9) | -0.1 (1.0) | 0.6 |
| Maternal characteristics |  |  |  |  |  |  |  |  |  |  |
| Schooling at child age 7/8 y, % |  |  |  |  |  |  |  |  |  |  |
| Matriculated | 27 | 0.5 | 30 | 28 | 0.7 | 31 | 0.9 | 35 | 30 | 0.2 |
| Marriage/union status, % |  |  |  |  |  |  |  |  |  |  |
| In union | 36 | 0.3 | 41 | 39 | 0.4 | 43 | 0.03 | 35 | 35 | 0.9 |
| Household characteristics |  |  |  |  |  |  |  |  |  |  |
| SES asset quintile at child age 7/8 y, % |  |  |  |  |  |  |  |  |  |  |
| 1 (lowest) | 23 | 0.07 | 12 | 18 | 0.09 | 26 | 0.1 | 24 | 24 | 0.007 |
| 2 | 19 |  | 20 | 21 |  | 23 |  | 17 | 18 |  |
| 3 | 29 |  | 32 | 29 |  | 24 |  | 32 | 28 |  |
| 4 | 16 |  | 22 | 23 |  | 17 |  | 14 | 20 |  |
| 5 (highest) | 13 |  | 14 | 8 |  | 10 |  | 19 | 10 |  |
| Adolescent Trajectories |  |  |  |  |  |  |  |  |  |  |
| **informal activity, %** |  |  |  |  |  |  |  |  |  |  |
| Decreasing over time |  |  | 92 | 93 | 0.5 |  |  | 92 | 95 | 0.1 |
| Increasing over time |  |  | 8 | 7 |  |  |  | 7 | 5 |  |
| **Organized sports participation, %** |  |  |  |  |  |  |  |  |  |  |
| Males |  |  |  |  |  |  |  |  |  |  |
| Consistently low |  |  | 82 | 82 | 0.7 |  |  |  |  |  |
| Decreasing from adequate to low |  |  | 7 | 6 |  |  |  |  |  |  |
| Increasing from low to adequate |  |  | 10 | 12 |  |  |  |  |  |  |
| Females |  |  |  |  |  |  |  |  |  |  |
| None |  |  |  |  |  |  |  | 89 | 89 | 0.9 |
| Consistently some |  |  |  |  |  |  |  | 11 | 11 |  |
| **Walking to and from school, %** |  |  |  |  |  |  |  |  |  |  |
| Consistently 150 min/week |  |  | 79 | 84 | 0.1 |  |  | 79 | 73 | 0.09 |
| Consistently 300 min/week |  |  | 21 | 16 |  |  |  | 21 | 27 |  |
| **Overall physical activity ^e^, %** |  |  |  |  |  |  |  |  |  |  |
| Decreasing activity over time and do not walk to school |  |  | 21 | 22 | 0.7 |  |  | 36 | 29 |  |
| Decreasing activity over time but consistently walk to school |  |  | 50 | 51 |  |  |  | 48 | 56 |  |
| Consistently more active and consistently walk to school |  |  | 30 | 27 |  |  |  | 16 | 15 |  |
| **Sedentary behavior, %** |  |  |  |  |  |  |  |  |  |  |
| Males |  |  |  |  |  |  |  |  |  |  |
| Consistently low |  |  | 66 | 83 |  |  |  |  |  |  |
| Consistently high |  |  | 15 | 6 |  |  |  |  |  |  |
| Increasing from low to high |  |  | 19 | 11 |  |  |  |  |  |  |
| Females |  |  |  |  |  |  |  |  |  |  |
| Initially low |  |  |  |  |  |  |  | 88 | 94 | 0.01 |
| Consistently high |  |  |  |  |  |  |  | 12 | 6 |  |
| **School-night sleep, %** |  |  |  |  |  |  |  |  |  |  |
| ≤ 8 hours/night |  |  | 69 | 69 | 0.9 |  |  | 68 | 64 | 0.3 |
| ≥ 9 hours/night |  |  | 31 | 31 |  |  |  | 32 | 36 |  |
| Young-adult characteristics (18 y) |  |  |  |  |  |  |  |  |  |  |
| BMI, kg/m2 |  |  | 20.7 (3.8) | 20.1 (2.6) | 0.02 |  |  | 23.5 (4.9) | 23.2 (4.4) | 0.2 |
| Waist- circumference |  |  | 71.2 (8.3) | 72.3 (6.6) | 0.09 |  |  | 75.4 (17.0) | 75.7 (10.9) | 0.8 |

^a^ Black-African participants who did not have movement behavior data at ≥2 timepoints and were therefore not included in adolescent trajectories and excluded from the analysis.

^b^ P-value for comparison of Black-African participants excluded from analysis to the participants (638 male and 699 female) who were included in adolescent trajectories and had a BMI or waist circumference data at age 18 years.

^c^ Participants who had anthropometric data (BMI, waist circumference) at age 18 years, but not DXA body composition data (lean mass, fat mass, percent fat).

^d^ Participants who had both anthropometric data and DXA body composition data at age 18 years).

^e^ Multi-trajectory groups made up of informal activity, organized sports and walking to and from school trajectories representing overall physical activity pattern.
